# Nef does not contribute to replication differences between R5 pre-AIDS and AIDS HIV-1 clones from patient ACH142

**DOI:** 10.1186/1742-4690-5-42

**Published:** 2008-05-29

**Authors:** Kevin C Olivieri, Robert M Scoggins, Brooks Broderick, Maria LC Powell, Melissa A Alexander, Marie-Louise Hammarskjöld, David Rekosh, David Camerini

**Affiliations:** 1 Department of Microbiology and Myles H. Thaler Center for AIDS and Human Retrovirus Research, University of Virginia, Charlottesville, VA 22908, USA; 2 Department of Molecular Biology and Biochemistry, Center for Immunology and Center for Virus Research, University of California, Irvine, CA 92697-3900, USA

## Abstract

AIDS-associated, CCR5-tropic (R5) HIV-1 clones, isolated from a patient that never developed CXCR4-tropic HIV-1, replicate to a greater extent and cause greater cytopathic effects than R5 HIV-1 clones isolated before the onset of AIDS. Previously, we showed that HIV-1 Env substantially contributed to the enhanced replication of an AIDS clone. In order to determine if Nef makes a similar contribution, we cloned and phenotypically analyzed *nef *genes from a series of patient ACH142 derived R5 HIV-1 clones. The AIDS-associated Nef contains a series of residues found in Nef proteins from progressors [1]. In contrast to other reports [1-3], this AIDS-associated Nef downmodulated MHC-I to a greater extent and CD4 less than pre-AIDS Nef proteins. Additionally, all Nef proteins enhanced infectivity similarly in a single round of replication. Combined with our previous study, these data show that evolution of the HIV-1 *env *gene, but not the *nef *gene, within patient ACH142 significantly contributed to the enhanced replication and cytopathic effects of the AIDS-associated R5 HIV-1 clone.

## Background

The *nef *gene of HIV-1 plays a pivotal role in the pathogenesis of AIDS [4-8]. For example, patients infected with *nef*-deleted HIV-1 exhibited much slower progression to AIDS [6,9-11]. The *nef *gene is important for viral replication in mature T cells [12-16] and macrophages [14,17-19]. When thymocytes are infected, Nef plays a role in increasing the cytopathic nature of the virus [20-24]. The importance of Nef is further corroborated by observations of immune dysfunction in *nef*-transgenic mice [25-28].

Several functions have been assigned to Nef although the role of each in disease progression has not been firmly established (for reviews see: [21,29-37]). We chose to focus on Nef's abilities to downmodulate CD4 [38] and cell surface MHC-I A and B molecules [39,40], and its ability to enhance viral infectivity [12,41,42]. These functions have been well studied by several labs and in various cell types and systems [17,43-50]. Nef mediated enhancement of infectivity may be due to Nef downmodulation of cell surface CD4, allowing more efficient Env incorporation into HIV-1 particles [51,52]. Enhancement of infectivity may also occur when Nef is present in CD4 negative producer cells [12,53-56]. In this case, enhancement appears to act at a post-entry, pre-integration step in the viral life cycle [57,58] and may be related to interaction of the viral pre-integration complex with the actin cytoskeleton [59]. Downmodulation of MHC-I A and B molecules protects cells from lysis by HIV-1 specific cytotoxic T cells [40]. The ability to avoid the immune system may be important in establishment and maintenance of infection.

Kirchhoff and colleagues compared the predicted amino acid sequences of Nef proteins from progressors with those of non-progressors and found that certain residues characterize Nef sequences from each type of patient [1]. When compared to non-progressor Nefs, progressor Nefs were better able to downmodulate CD4 and less able to downmodulate MHC-I molecules, and also may have an increased ability to enhance HIV-1 infectivity [1,2].

## Results

Previously, we demonstrated that the ACH142 AIDS clone *E11 was better able to replicate and cause cytopathic effects in human fetal thymus-liver grafts implanted in severe combined immune deficient mice (SCID-hu thy/liv mice), than the pre-AIDS clones, 8G9 and 32D2 [60]. In an analysis parallel to this study, we examined the phenotypes of the *env *genes from these clones and determined that the AIDS associated *env *likely contributed to the observed replication differences between the AIDS clone and the pre-AIDS clones [61]. In order to determine if *nef *made a similar contribution, ACH142 *nef *genes were amplified from PHA-activated PBMC infected with the HIV-1 clones ACH142-*E11, 32D2, and 8G9. The one kilo base *nef/*LTR products were gel purified and inserted into the pGEM-T vector (Promega). Six *E11, six 32D2 and three 8G9 full-length *nef *genes were sequenced.

**Figure 1:**
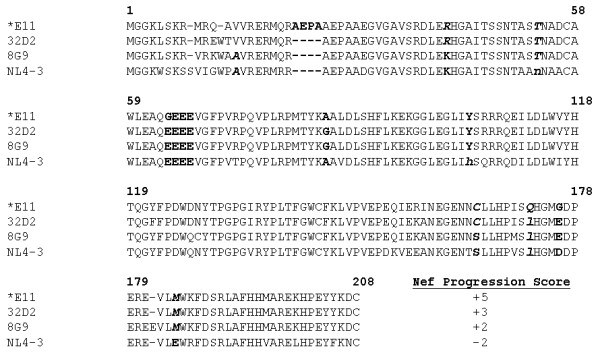
The R5 AIDS HIV-1 clone ACH142-*E11 Nef has a higher progression score than the Nefs from two patient ACH142 derived pre-AIDS R5 HIV-1 clones, 32D2 and 8G9. A Clustal W alignment of predicted Nef amino acid sequences is shown. Gaps in the alignment are represented by a dash. Bold amino acid residues represent changes of interest between isolates. Numbers above the alignment represent the amino acid position in this alignment. Bold, italicized residues were used to calculate the Nef progression score according to the method of Kirchhoff *et al *[1]. Upper case bold italicized letters indicate residues that are more common in progressors. Lower case bold italicized letters indicate residues that are more common in non-progressors.

Analysis of the predicted amino acid sequences of the consensus Nef proteins revealed a high degree of conservation among the patient ACH142 biological clones (Fig. 1). When compared to sequence/function studies reported in the literature, as reviewed in [31], no lack of function mutations could be found, but three interesting differences were revealed. The AIDS Nef protein contains the rare motif GEEE (amino acids 62–65), whereas the two pre-AIDS proteins contain the more common EEEE sequence at this position. This motif has been reported to be important in MHC-I downmodulation. The *E11 sequence also has a significant lengthening of the N-terminal portion of Nef caused by repetition of the four amino acid sequence, AEPA (amino acids 23–26). Using the analysis of Kirchhoff *et al*, we calculated the Nef progression score of each ACH142 Nef. A +1 score was assigned for residues characteristic of progressors and a -1 score was assigned for those commonly found in non-progressors at the positions denoted by bold italic symbols (Fig. 1). This number therefore reflects the degree of similarity between each ACH142 Nef sequence and Nefs from progressors or non-progressors at particular amino acid positions. We found that the AIDS associated *nef*, *E11, is more similar to progressor Nef sequences with a Nef progression score of +5 than are the pre-AIDS Nefs from the same patient which had Nef progression scores of +2 and +3.

To elucidate the significance of these differences and to assign phenotypes to each Nef protein, we inserted each consensus *nef *gene into an actin promoter driven expression vector (pA-*nef*). Next we analyzed the ability of each Nef to downmodulate cell surface CD4 and MHC-I A2 molecules on the T lymphoblastoid cell line, SupT1. Electroporation and flow cytometric analysis of SupT1 cells bearing pA-*nef *expression vectors was done as previously described [50]. Two μg of pCMV-*EGFP *and 10 μg of each pA-*nef *expression plasmid, or empty pA vector were introduced into SupT1 cells by electroporation. The cells were then plated in 10 cm dishes and cultured for 24 hours. Subsequently, the cells were incubated with CD4-PerCP and MA2.1-PE monoclonal antibodies to detect CD4 and the A2 allele of MHC-I by flow cytometry. Data were collected with a FACSCalibur instrument and the GFP+ population was analyzed for CD4 and MHC-I surface expression with CellQuest software.

**Figure 2:**
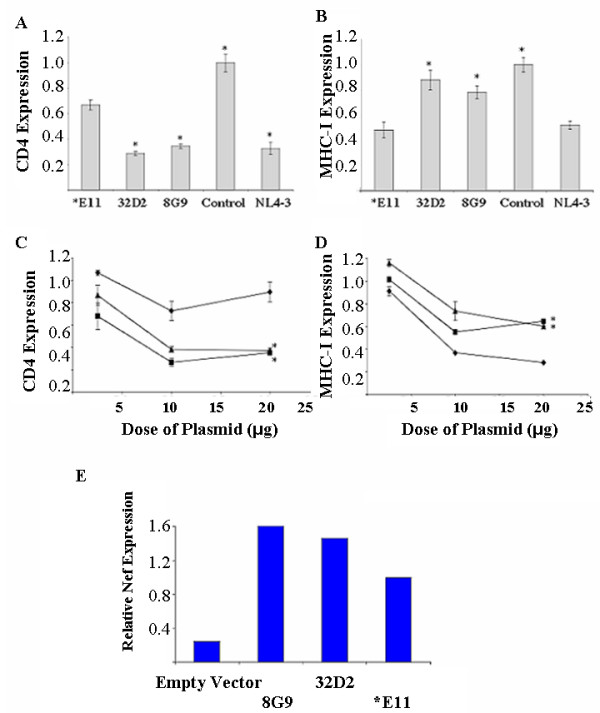
The R5 AIDS Nef from HIV-1 clone *E11 does not down regulate CD4 more than the two pre-AIDS alleles from 32D2 and 8G9, but does downregulate MHC Class I more than the pre-AIDS Nefs. SupT1 cells were electroporated with 10 μg of pA-Nef expression vectors and 2 μg of pCMV-EGFP expression vector. Cells were analyzed by flow cytometry 24-hrs post-electroporation. The fraction of control levels of cell surface CD4 (**A **and **C**) or MHC Class I A2 (**B **and **D**) expression in GFP+ cells are reported for each allele. **C **and **D **2.5, 10 or 20 μg of pA-*E11 Nef (diamonds), pA-32D2 Nef (squares), or pA-8G9 Nef (triangles) were  transferred to SupT1 cells by electroporation. The average of eight transfections for **A **and **B **or two transfections for **C **and **D **is shown. Error bars represent the standard errors of the mean. Samples denoted with asterisks were significantly different from the *E11 sample as determined by the Student's unpaired t-test (**A **and **B**) or by the Student's paired t-test (**C **and **D**). **E **Twenty μg of pA-Nef expression vectors were used for electroporation of SupT1 cells with 2 μg of pCMV-EGFP. Cells were lysed in sample buffer and analyzed by SDS-PAGE and western blot. The blot was probed with a polyclonal rabbit anti-Nef serum followed by ^125^I-Protein A. The blot was then analyzed by phosphorimager and quantitated using ImageQuant software. Results from a representative experiment of three experiments performed are shown.

Expression of consensus *nef *genes from the two pre-AIDS clones, 8G9 and 32D2, induced the highest level of CD4 downmodulation, similar to that of the NL4-3 *nef *gene (Fig. 2). The late stage *E11 consensus *nef *gene induced significantly less CD4 downmodulation (p < 0.0001 by Student's t-test). In contrast, the ability to downmodulate MHC-I A2 molecules was similar for *E11 and NL4-3 *nef *genes, while the *nef *genes from the earlier ACH142 clones exhibited significantly less MHC-I downmodulation (p < 0.003 by Student's t-test). When increasing doses of Nef expression plasmid were used in electroporation of SupT1 cells, this difference was heightened. In no case was the *E11 Nef better able to downmodulate CD4 than the two earlier clones' Nefs or NL4-3 Nef. Likewise, in no case were the two pre-AIDS Nef alleles better able to downmodulate MHC-I A2 molecules than the *E11 AIDS Nef. Nef expression levels were similar for all three patient ACH142 derived *nef *genes. The 8G9 and 32D2 Nefs were expressed at 1.5 and 1.6 times higher levels than the *E11 Nef respectively, as determined by radiometric quantitation of a Nef immunoblot (Fig. 2E).

**Figure 3:**
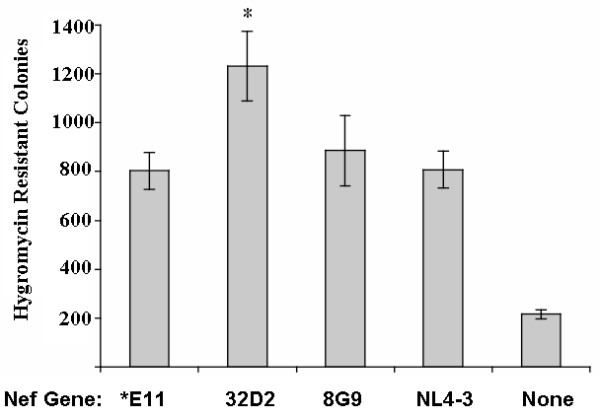
The R5 pre-AIDS HIV-1 clone 32D2 Nef protein enhances infectivity more than the R5 AIDS allele. The 5BD.1 HIV-1 vector packaging cell line was co-transfected with pTR167 ΔNef (5 μg), pCMV-Tat (2 μg) and *E11, 32D2, NL4-3, or 8G9 pCMV Nef plasmid (2.5 μg). Three days post-transfection, 100 μl of the cell supernatants were used to infect 2 × 10^5 ^HeLa CD4 cells in the presence of 8 μg/ml DEAE-dextran. Viral vectors and cells were incubated together at 37°C for 24 hours. At that time, infectious media was removed and replaced with IMDM plus 10% BCS. At 48 hours post-infection, IMDM + 10% BCS and hygromycin (200 μg/ml) was added. After two weeks of selection, the resultant colonies were stained with crystal violet and manually counted. The average of nine infections from two different viral vector stocks for each Nef is shown. Error bars represent standard errors of the mean (SEM). Asterisked 32D2 samples were significantly different from each of the three other Nef positive samples by the Student's unpaired t-test.

The contribution of each consensus *nef *gene to HIV-1 infectivity was determined using the CD4 negative HIV-1 packaging cell line, 5BD.1 and the hygromycin resistance gene-bearing HIV-1 derived vector, TR167 [56]. Cells were co-transfected with pTR167 Δ*nef *(5 μg), pCMV-Tat (2 μg) and either the *E11, 32D2, 8G9 or NL4-3 pCMV-*nef *expression vector (5 μg) to produce hygromycin resistance-transducing HIV-1 vector particles. Vector stocks were used to infect HeLa-CD4 cells; after two weeks of selection with hygromycin, colonies were stained with crystal violet and counted (Fig. 3). All *nef *genes studied here significantly enhanced the infectivity of the vector when compared to *nef *negative vectors (p < 0.001 by Student's t-test). The 32D2 pre-AIDS *nef *enhanced infectivity significantly more than the *E11 AIDS *nef *(p < 0.01 by Student's t-test). Similar infectivity enhancement was mediated by the 8G9 pre-AIDS *nef*, the *E11 AIDS *nef *and NL4-3 *nef*. Nearly identical results were observed when each patient *nef *was used to complement vectors created with *env *genes from the same HIV-1 biological clone (data not shown).

## Discussion

Our results suggest that the AIDS associated *nef *gene studied here does not significantly contribute to the enhanced replication and cytopathic effects of the AIDS associated *E11 R5 HIV-1 clone for the following three reasons. It is highly conserved at almost all known sites within the Nef sequence that are implicated in functional interactions. It does not downmodulate CD4 to a greater extent than pre-AIDS Nefs, nor does it more greatly enhance infectivity in a single round assay compared to the pre-AIDS Nefs from the same patient. Combined with our previous study, we conclude that Env, but not Nef contributes to the enhanced replication of the R5, AIDS-associated HIV-1 clone ACH142-*E11 compared to two pre-AIDS R5 HIV-1 clones derived from the same patient [61].

Previous studies indicated that Nef proteins with high progression scores had enhanced ability to downmodulate CD4, reduced ability to downmodulate MHC-I and increased ability to enhance HIV-1 replication compared to Nef proteins with lower progression scores [1-3]. The Nef proteins of patient ACH142 derived R5 HIV-1 clones displayed a chronological increase in Nef progression score as predicted, but the phenotype of these genes differed from the predicted phenotypes described above. The AIDS associated Nef protein studied here had a higher progression score (+5) than the pre-AIDS Nef proteins derived from the same patient (+2 and +3), but did not show an increased ability to downmodulate CD4 or to enhance infectivity. Moreover, the AIDS associated Nef protein had greater ability to downmodulate MHC-I A2 molecules. This is likely not explained by differences in Nef expression because the *E11 Nef downmodulated MHC-I to a greater extent despite being expressed at a slightly lower level. One explanation for the discrepancy between our results and those previously reported by others may be that *nef *genes from CXCR4-tropic (X4) HIV-1 isolates have the phenotypes previously reported [2,3] but *nefs *from R5 HIV-1 clones show the phenotypes demonstrated here. Most of the progressor *nefs *used in previous studies were likely derived from X4 HIV-1 because patients with X4 HIV-1 progress to AIDS more rapidly. In contrast, all three *nef *genes studied here were derived from patient ACH142, who never developed X4 HIV-1 [60,61]. More analyses of *nef *genes from R5 HIV-1 clones derived from progressors are needed to test the generality of our observations.

Previous studies have shown that X4 HIV-1 isolates are more sensitive to neutralization by soluble CD4 than R5 HIV-1 [62-64] and that X4 HIV-1 clones incorporate less Env into their virions when cellular CD4 is not downmodulated than R5 HIV-1 clones [52]. It is therefore likely that downmodulation of CD4 has a greater impact on X4 HIV-1 replication than on R5 HIV-1 replication. R5 HIV-1 Nef may therefore have a greater positive effect on viral replication by down-modulating cell surface MHC-I molecules and thereby protecting infected cells from lysis by anti-HIV-1 cytotoxic T lymphocytes.

Moreover, because R5 HIV-1 clones preferentially infect effector memory T cells and macrophages while X4 HIV-1 clones preferentially infect naïve T cells, *nef *genes in each type of HIV-1 may evolve over the course of an infection to better enhance replication in each respective cell type. Determining whether such an evolution occurs may allow us to find specific Nef interactions that occur preferentially in macrophages or in memory or naïve T cells. In particular, Nef's ability to activate T cells may be more essential for X4 HIV-1, since naïve T cells are less easily activated than memory T cells.

Our data lend support to the notion that Nef cannot evolve over the course of disease to enhance all of its functions. This is true for our study in which an AIDS-associated Nef was reduced in its ability to downmodulate CD4 and enhanced in its ability to downmodulate MHC I and is also true for other studies where the reverse was found. This supports a paradigm whereby increased Nef mediated downmodulation of CD4 or MHC Class I molecules correlates with a loss in the other function. This paradigm may be created by structural constraints that limit the ability of Nef to perform both functions optimally or by competition for limiting cellular factors necessary for both processes. Determination of these constraints and/or factors will shed light on the role of Nef in HIV-1 replication and pathogenesis.

## Competing interests

The authors declare that they have no competing interests.

## Authors' contributions

KCO carried out most of the experiments and wrote the paper, RMS and BB cloned the nef genes and initiated the calculation of Nef progression scores, MP helped perform the downmodulation and infectivity experiments, MAA developed the protocol for the downmodulation assays and advised on their use, DC, M-LH and DR designed the study and DC edited and revised the manuscript.
